# Lifestyle Behaviours in Pre-Schoolers from Southern Spain—A Structural Equation Model According to Sex and Body Mass Index

**DOI:** 10.3390/nu16213582

**Published:** 2024-10-22

**Authors:** Gracia Cristina Villodres, Rosario Padial-Ruz, José-Antonio Salas-Montoro, José Joaquín Muros

**Affiliations:** 1 Department of Didactics of Corporal Expression, Faculty of Education, University of Granada, 18071 Granada, Spain; gcvillodres@ugr.es (G.C.V.); rpadial@ugr.es (R.P.-R.); 2 Department of Physical Education and Sports, Faculty of Sports Sciences, University of Granada, 18071 Granada, Spain; salasmontoro@ugr.es

**Keywords:** screen time, sleep time, physical fitness, Mediterranean diet, eating behaviours, body mass index, childhood

## Abstract

Objectives: The present study aimed to examine the relationship between screen time (ST), sleep time (SLT), physical fitness (PF), Mediterranean diet (MD) adherence, eating behaviours, and body mass index (BMI) in a sample of pre-schoolers from Granada (Spain). In order to address this aim, an explanatory model was developed to examine existing relationships between ST, SLT, PF, MD, pro-intake (PRO-I) and anti-intake (ANT-I) behaviours, and BMI. Further, the proposed structural model was examined via multi-group analysis as a function of sex and BMI. Methods: A cross-sectional study was conducted with 653 three- to six-year-old pre-schoolers attending 18 different schools invited to take part in the present study. Structural equation modelling (SEM) was employed to analyse relationships between study variables as a function of sex and BMI. Results: SEM analysis revealed negative associations between ST and PF (*p* < 0.005), ST and MD adherence (*p* < 0.005), ST and SLT (*p* < 0.005), MD adherence and ANT-I behaviours (*p* < 0.005), and MD adherence and BMI (*p* = 0.033). In contrast, positive associations emerged between SLT and MD adherence (*p* < 0.005), and PRO-I behaviours and BMI (*p* < 0.005). SEM revealed differences according to sex and BMI. Conclusions: The study highlights significant relationships between lifestyle behaviours and physical and dietary outcomes in pre-schoolers from southern Spain, with variations based on sex and BMI. These findings suggest the need for interventions aimed at reducing ST and promoting better sleep, PF, and dietary habits in order to limit weight-related and general health risks in pre-schoolers from southern Spain.

## 1. Introduction

Obesity is a chronic and complex condition characterised by excessive fat accumulation that can negatively impact health. It can influence quality of life, including aspects such as sleep and mobility. Likewise, obesity is associated with increased risk of type 2 diabetes, heart disease, and certain cancers, and may affect bone health and reproduction. Additionally, childhood obesity confers major risks of excess and premature morbidity and mortality in both sexes [1]. This is supported by the World Health Organization (WHO) [2], which also reported in 2022 that 37 million children under the age of five were overweight.

In Spain, in 2020, one in three children between two and 17 years old was overweight, whilst one in ten was obese [3]. Moreover, the most recent data gathered by the ALADINO study in 2019 [4,5] reveal that 23.3% of Spanish schoolchildren are considered overweight, whilst 17.3% are considered obese. This means that a total of 40.6% of the child population is affected by excess bodyweight. Girls are more likely to be overweight (24.7%) than boys (21.9%), whilst the prevalence of obesity is notably higher in boys (19.4%) than in girls (15.0%). When compared with other nations covered by the COSI strategy, Spain ranks second with regards to prevalence of excess weight and sixth with regards to obesity prevalence [6]. Further, according to the PASOS study [7], 33.4% of the adolescent population is overweight or obese. These statistics underline the urgency of addressing this issue in Spain, making it necessary to intervene from an early age, including with the pre-school population, and curbing the rise in overweight and obesity during the school years and throughout adolescence.

Excess weight, along with the chronic non-communicable diseases associated with it, is largely preventable. It is crucial to understand the scale of this issue and the factors that contribute to it. This knowledge is essential for the design, implementation, and evaluation of strategies and interventions aimed at mitigating this problem [5]. The main cause of childhood obesity is energy imbalance in which energy intake surpasses energy expenditure, with genetics also playing a significant role regarding predisposition towards weight gain [8]. Nevertheless, for most obese children, weight gain cannot be attributed to a single genetic or endocrine factor [9]. This suggests that lifestyle factors are the dominant contributors to the early development of obesity. A systematic review and meta-analysis by Poorolajal et al. [10] indicated that factors associated with higher childhood body mass index (BMI) were insufficient physical activity, poor diet, inadequate sleep, and engaging in more than two hours of screen time per day.

Limiting screen time (ST) (<1 h), adequate sleep duration (SLT) (10–13 h), good physical fitness (PF) (adequate cardiorespiratory capacity, strength, speed/agility and balance, and adequate Mediterranean diet (MD) adherence (fruits, vegetables, whole grains, legumes, nuts, olive oil and lean proteins, particularly fish, and limited processed foods) play a crucial role in overall quality of life in children [11,12,13]. Further, higher MD adherence, physical activity engagement, and good PF are associated with a heathy weight status in children [14].

In contrast, decreased physical activity and increased sedentary behaviour are two of the main factors that have contributed to the rise in obesity [15]. Specifically in Spain, it has been observed that 26.3% of schoolchildren spend two hours or more watching TV or using electronic devices during the week, with this being true for 75.4% of schoolchildren on weekends [4]. This increase in ST may be associated with greater reductions in SLT, with one in three children under five years of age reported to have poor sleep quality at night [16]. At the same time, only 37.1% of Spanish schoolchildren consume fresh fruit daily, and just 13.4% eat vegetables every day. Moreover, 53.9% consume pastries, commercial juices, or milkshakes as part of their breakfasts. Also of concern is the frequent consumption of certain foods that should only be part of a schoolchild’s diet occasionally, such as biscuits, cakes, or pastries, with 25.3% of schoolchildren reporting eating such foods more than four times a week [4].

In light of the factors discussed above, pre-schooler’s ST is negatively associated with their consumption of vegetables and fruits, and positively associated with their consumption of snacks and sugary drinks [17]. In this sense, insufficient physical activity and excessive ST in this population have been found to be linked with less healthy patterns of food and beverage consumption [18]. Further, Spanish children aged three to twelve years with more sedentary lifestyle have also been reported to exhibit shorter duration and poorer quality sleep, lower MD adherence, and poorer PF, with such children also having a less healthy weight [19,20]. In this regard, due to the rise in new technologies, children can easily be attracted to technological devices, leading them to engage in more sedentary behaviour and less physical activity, which in turn causes them to miss opportunities for developing PF. Socially, this behaviour is often accompanied by the consumption of unhealthy snacks [17], which can have a negative influence on children’s BMI and risk of being overweight or obese.

With regards to eating habits, not only is it necessary to follow a healthy diet but, also, food preferences play a significant role at this stage. Eating behaviours are formed in the early years of childhood; however, such behaviours can evolve over time based on individual experiences [21]. Jimeno-Martínez et al. [22] reported that Spanish pre-schoolers with a higher BMI reported higher pro-intake (PRO-I) scores, specifically with regards to the “food responsiveness”, “emotional overeating”, and “enjoyment of food” subscales. Further, higher ST and shorter SLT are associated with poorer eating behaviours such as “mindless eating” or the inclination towards “reward foods” [23,24]. Along the same lines, pre-schoolers with higher anti-intake (ANT-I) behaviours reported lower fish consumption and more frequently consumed fast food [25], thus moving away from adequate MD adherence.

Moreover, existing studies highlight the importance of differentiating intervention factors as a function of sex and BMI and of considering the sociocultural differences at play [26]. In this sense, with regards to sex differences in children, boys generally spend more time playing video games, whilst girls engage more with social media and passive entertainment [27]. Girls also tend to have stricter sleep routines, leading to better sleep quality, whereas boys often use screens late at night, resulting in shorter and poorer sleep [28]. In the case of physical fitness, boys are more involved in competitive sports, whilst girls lean towards non-competitive activities such as dance, with this trend being largely influenced by social barriers [29]. With regards to dietary habits, girls typically choose healthier foods and exhibit better emotional control, whereas boys are more likely to consume unhealthy snacks [30].

Similarly, regarding BMI differences in children, compared to those with a healthy BMI, children with a higher BMI tend to have more ST, characterised by high video games and passive entertainment use [31]. An unhealthy BMI is also linked to shorter and poorer quality sleep due to increased ST before bed, whereas children with a healthy BMI engage in more regular and better sleep [32]. With regards to PF, children with a healthy BMI are more likely to engage in vigorous activities and belong to sports clubs, whilst those with an unhealthy BMI tend to be more sedentary [33]. In terms of diet, children with a healthy BMI consume more fruits, vegetables, and MD staples, whilst those with an unhealthy BMI favour processed foods and sugary drinks, and exhibit more emotional eating behaviours [34].

In physiological terms, positive correlations have been demonstrated between BMI and triglycerides (TG) and low-density lipoprotein cholesterol (LDL-C) from as early as three years old [35]. Consequently, BMI correlates with future obesity risk and metabolic issues such as insulin resistance and dyslipidaemia [36]. Further, previous studies have shown that a BMI classified as overweight (above the 85th percentile) or obese (95th percentile) in early childhood is linked with fat accumulation, waist circumference, and adiposity [37], making it a useful predictor even when body composition cannot be directly measured. Additionally, BMI is easily measured, making it a useful variable for large-scale studies. It is also especially important during the pre-school years, as it can predict future weight trajectories [38] and highlight the need for early intervention.

A more active lifestyle adopted in the first years of life often persists into later life, making the period between three to six years of age critical for beginning to establish a healthy lifestyle [38]. Despite this, pre-schoolers have been less extensively studied than older age groups when it comes to the health impact of certain lifestyle habits [39]. This is primarily due to challenges associated with assessing this population and restricted access to potential participants, particularly in health-related areas, such as parental concerns around education, participation burden, lack of trust, general research concerns, informational- and consent-related challenges, and relational issues [40]. These challenges must, therefore, be overcome in order to better examine this population.

The topic addressed by the manuscript has been widely studied, however, no previous studies have examined the wider set of potential lifestyle determinants together. This includes eating behaviours, with a relevant questionnaire being recently adapted for use with Spanish samples [22], although its application to pre-schoolers is highly innovative. Another question to address is whether relationships exist between healthy lifestyle habits and BMI at such early ages and whether sex plays a role. Based on the existing literature, an alternative hypothesis of the present study is that, from very early ages, relationships emerge between healthy lifestyle habits and BMI and, consequently, health. Likewise, it is expected that sex will impact this relationship.

Thus, the present study aimed to examine the relationship between ST, SLT, PF, and MD adherence, eating behaviours, and BMI in a sample of pre-schoolers from Granada, Spain. In order to address this aim, an explanatory model was developed to examine existing relationships between ST, SLT, PF, MD, ANT-I and PRO-I, and BMI. Further, the proposed structural model was examined via multi-group analysis as a function of sex and BMI.

## 2. Materials and Methods

### 2.1. Participants

A cross-sectional study was designed. A sample of 653 pre-schoolers aged three to six years was recruited to the study. A total of 2250 children from 18 schools (Granada, Spain) were invited to take part in the present study between April 2023 and April 2024. Participating schools were selected through convenience due to them being easy to access. In total, 336 girls (51.5%) and 317 boys (48.5%) with an average age of 4.78 years ± 0.93 years participated in the study. The main inclusion criterion referred to pre-school children who do not suffer from food allergies or physical or psychiatric conditions. In order to address this, the statement “observations” was included at the end of the questionnaire, inviting open responses and enabling parents to disclose information regarding any physical, psychiatric, or dietary conditions that could affect outcomes. No parents responded to this question, suggesting that no participants fell into this category.

### 2.2. Procedure

**Figure 1:**
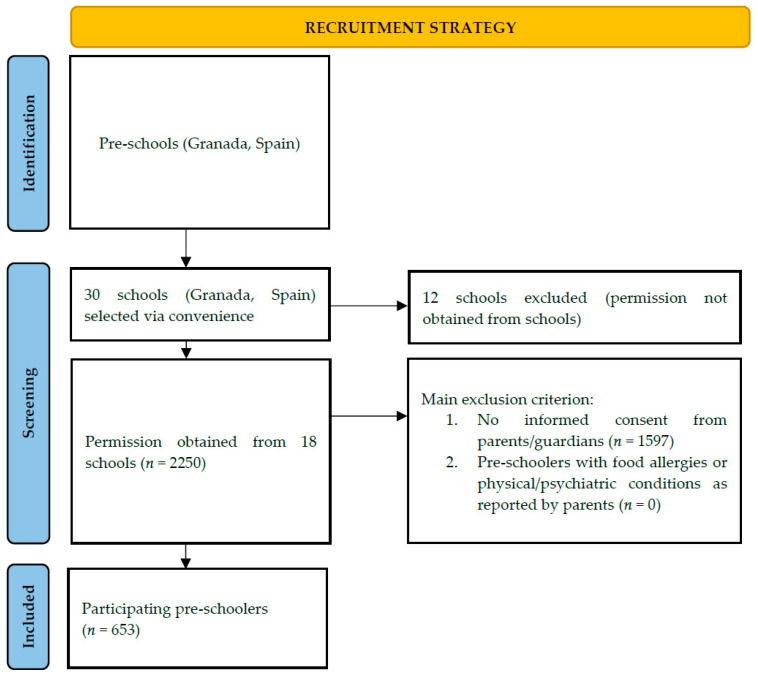
Recruitment strategy.

The recruitment strategy is presented in Figure 1. In order to access the study target population of pre-school children, 30 schools were selected which had a pre-existing relationship with researchers. Of these 30 schools, directors at 12 schools declined to participate. After receiving permission from 18 schools (*n* = 2250), informed consent was requested from the parents or legal guardians of the children belonging to each of the schools. In this way, parents were informed about the study aims, design, and procedure. Following the receipt of written informed consent (*n* = 653), a questionnaire was sent out for voluntary completion by parents or legal guardians on behalf of their children. The Google Drive^®^ application (Alphabet, Mountain View, CA, USA) was used. Ethical principles of the Declaration of Helsinki for Medical Research were followed, and ethical approval was granted by the Ethics Committee of the University of Granada 2794/CEIH/2022.

### 2.3. Instruments

The latest version of the Mediterranean Diet Quality Index (KIDMED) was used to evaluate children’s MD adherence [41]. This comprises a total of 16 items which canvass binary responses (“yes” or “no”). Four items are negatively framed, and so positive responses are scored as −1. Conversely, the remaining items are positively framed, with affirmative responses being scored as +1 and negative responses receiving a score of 0. As a result, potential overall final scores range from −4 to 12.

PF was evaluated using the latest Spanish edition of the International Fitness Scale (IFIS) [42]. This questionnaire comprises five items, with each being measured on a five-point scale ranging from 1 (very poor) to 5 (very good). Items assess essential aspects of self-perceived fitness (1: cardiorespiratory fitness; 2: muscular fitness; 3: speed-agility; 4: balance; and 5: overall PF). Individual scores for each item are summed to produce an overall fitness score. An average score is calculated from the five items to obtain an overall test score.

The latest Spanish edition of the Child Eating Behaviour Questionnaire (CEBQ) was employed to evaluate children’s eating behaviours [22,43]. This comprises a total of 36 items that are rated along a five-point Likert scale. Items can be grouped into two dimensions, namely, pro-intake (PRO-I) and anti-intake (ANT-I). PRO-I refers to food responsiveness, emotional overeating, enjoyment of food, and desire to drink, whilst ANT-I refers to satiety responsiveness, slowness in eating, emotional undereating, and food fussiness.

Sociodemographic data were collected through an ad hoc questionnaire. Parents reported their children’s sex, date of birth, height, and weight. BMI was then calculated from height and weight (BMI = weight [kg]/(height [m])^2^). Age and sex standardized classifications of healthy weight, overweight, and obese were calculated in line with ranking percentiles outlined by Sobradillo et al. [44]. Overweight and obese groups were combined due to the low percentage of children reporting being overweight (4.9%) and obese (6.3%). Children’s parents reported the number of hours of nightly sleep on weekdays and weekends. Similarly, two items were added to examine the number of hours spent daily solely engaged in screen-based leisure activities (watching television, playing video games, using a mobile phone, using a computer, etc.) on weekdays and weekends. Hours spent in front of the screen in in-person or online classes were not considered. Overall summary scores were calculated from the mean number of hours reported over the seven days examined.

### 2.4. Statistical Analysis

**Figure 2:**
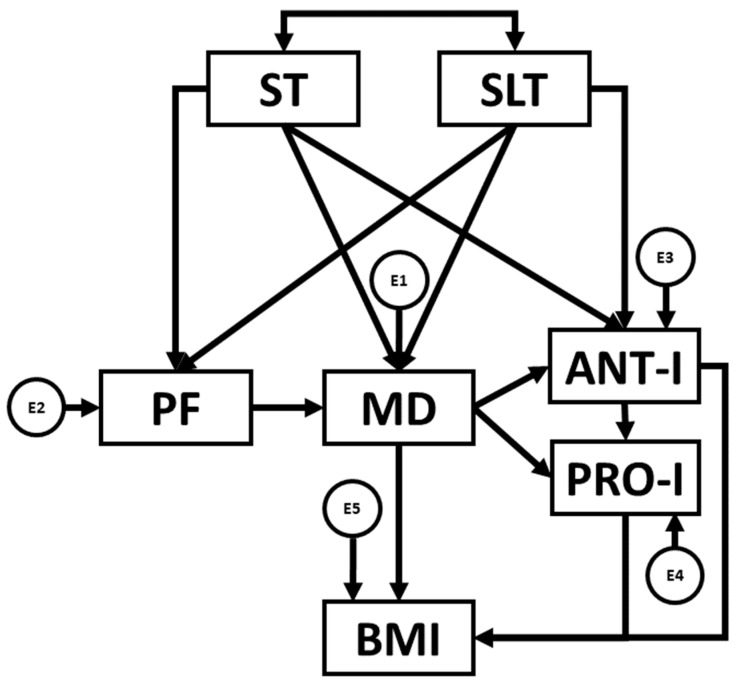
Structural equation model. Note: ST: screen time; SLT: sleep time; PF: physical fitness; MD: Mediterranean diet; ANT-I: anti-intake; PRO-I: pro-intake; BMI: body mass index; E1–E5: error terms.

Categorical data such as sex and BMI are represented as percentages. Mean and standard deviations are reported for all quantitative variables. Normality of the data was tested using the Kolmorov–Smirnov test. After verifying that data were not normally distributed, the data were analysed using the Mann–Whitney U test to compare two independent groups. Associations between quantitative variables were examined according to Spearman correlations (*p* < 0.05). All statistical analyses were performed using version 25 of IBM-SPSS^®^ for Windows. Then, structural equation modelling (SEM) was performed using IBM AMOS^®^ 24.0 software. SEM facilitates the establishment of connections between variables specified in a theoretical model (Figure 2). The model constructed in the present study comprised seven observed variables. Of these, five were endogenous (PF, MD, ANT-I and PRO-I and IMC) and two were exogenous variables (ST and SLT). Endogenous variables are represented within the developed model alongside their associated error terms, which are illustrated with a circle, whilst exogenous variables do not have associated error terms and are depicted via two-way arrows. SEM was employed to determine existing relationships between the variables included in the theoretical model as a function of sex (boy/girl) and BMI (healthy weight/overweight/obese).

In order to determine the extent to which the structural equation model (SEM) aligns with the observed data, several indices were utilized to assess model adequacy. Model fit should primarily be evaluated according to chi-square, with a non-significant *p*-value suggesting good fit. However, Byrne [45] argues that this statistic can be heavily impacted by sample size and so additional fit indices should also be considered. To this end, comparative fit (CFI), incremental fit (IFI), normalized fit (NFI), and Tucker–Lewis (TLI) indices were calculated. For model fit to be deemed acceptable, these indices should reflect values higher than 0.90, with values above 0.95 indicating excellent fit. Furthermore, root mean square error of approximation (RMSEA) was estimated, with values below 0.08 reflecting acceptable fit and values below 0.05 suggesting excellent fit.

#### Justification of Model Specification and Goodness of Fit Indices

The first structural equation model was built based on research presented above.

ST and SLT have been identified as key factors influencing physical health and eating behaviours. Increased ST, along with inadequate sleep, has been found to be associated with greater physical inactivity, lower adherence to healthy diets such as the MD, and less healthy eating behaviours [19,20]. For this reason, ST and SLT are entered in the first level of the model.

PF has been positively linked to greater MD adherence, due to the relationship between regular physical activity and increased awareness of the importance of proper nutrition for physical performance and overall health. This aligns with studies that highlight that physically active individuals tend to follow healthier dietary patterns [18]. For this reason, PF, MD adherence, and eating behaviours are included at the second level of the model in order to establish the influence of PF on MD adherence and, in turn, eating behaviours.

Finally, evidence suggests that adequate MD adherence, characterized by a balanced intake of fruits, vegetables, legumes, and healthy fats, has a positive impact on both eating behaviours and the maintenance of a healthy BMI, with diet being one of the main determinants of BMI [8].

Following specification of the model, directions of association between variables were determined in accordance with the Spearman correlation analysis. Following this, initial model fit was examined. Ultimately, model fit was refined until acceptable or excellent values were achieved for CFI, IFI, NFI, and TLI. These indices were selected due to their ability to provide comprehensive assessment of model fit. These indices are widely recognised in SEM literature as robust metrics that help to overcome the limitations of the chi-square statistic [45].

## 3. Results

Table 1 presents the descriptive characteristics of the study sample. With regards to sex, boys reported less SLT (10.29 ± 0.61 vs. 10.41 ± 0.70; *p* = 0.011) and scored lower for ANT-I (2.86 ± 0.56 vs. 2.94 ± 0.53; *p* = 0.019) and satiety responsiveness (2.75 ± 0.69 vs. 2.88 ± 0.64; *p* = 0.004) than girls. No further sex differences were observed for the remaining study parameters. With regards to BMI, relative to overweight or obese children, children with a healthy body weight reported higher scores for overall PF (4.30 ± 0.64 vs. 4.14 ± 0.65; *p* = 0.045) and cardiovascular fitness (4.08 ± 0.75 vs. 3.82 ± 0.71; *p* = 0.003) and lower scores for PRO-I (2.48 ± 0.53 vs. 2.74 ± 0.68; *p* = 0.003) and all of its subdimensions except desire to drink. Further, children with a healthy body weight reported higher scores for ANT-I (2.92 ± 0.56 vs. 2.77 ± 0.53; *p* = 0.023) and satiety responsiveness (2.84 ± 0.67 vs. 2.66 ± 0.67; *p* = 0.037) than overweight or obese children.

Table 2 presents correlation coefficients produced between study variables. ST was positively associated with ANT-I (r = 0.144; *p* < 0.01) and inversely associated with SLT (r = −0.219; *p* < 0.01), overall PF (r = −0.152; *p* < 0.01), and MD adherence (r = −0.262; *p* < 0.01). In contrast, SLT was positively associated with overall PF (r = 0.093; *p* < 0.05) and MD adherence (r = 0.235; *p* < 0.01), and inversely associated with ANT-I (r = −0.106; *p* < 0.01). Further, higher overall PF was positively associated with MD adherence (r = 0.113; *p* <. 05) and negatively associated with ANT-I (r = −0.160; *p* < 0.01). At the same time, MD adherence was inversely associated with ANT-I (r = −0.287; *p* < 0.01), with the latter also being inversely associated with PRO-I (r = −0.403; *p* < 0.01).

Given that a number of significant differences emerged according to sex and BMI, another SEM was constructed to better understand the relationship between study variables.

This SEM showed good fit indices for the multi-group analysis. The chi-square outcome was significant (*χ*^2^ = 16.9; *df* = 7; *p* = 0.018), meaning that this index does not support the goodness of fit of the model. Nonetheless, according to Marsh [46], this index cannot be interpreted in a standardised way due to its sensitivity to sample size. Thus, other standardised fit indices were used that are less sensitive to sample size [47]. In this way, NFI and TLI values were acceptable, being 0.948 and 0.902, respectively. IFI and CFI values were excellent, being 0.969 and 0.967, respectively. At the same time, the RMSEA value was also excellent, being 0.047.

**Figure 3:**
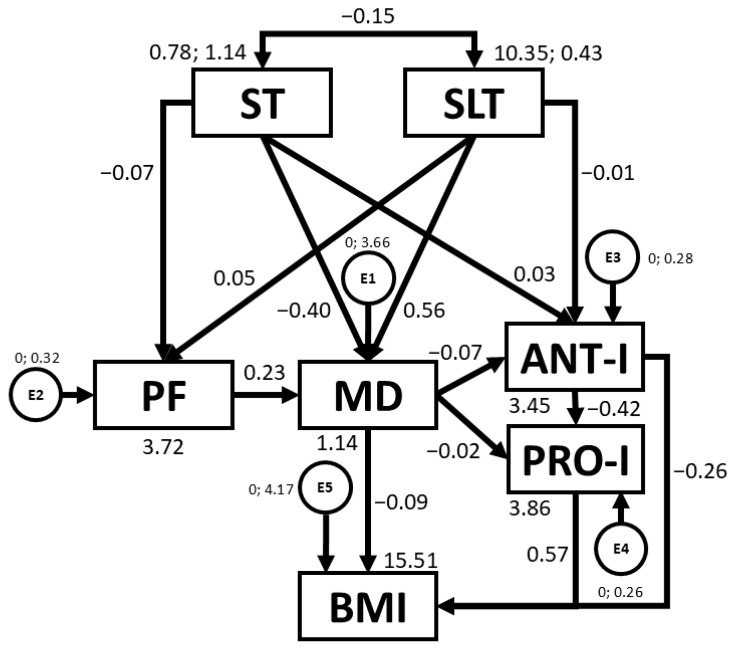
Structural equation model for the overall sample. Note: ST: screen time; SLT: sleep time; PF: physical fitness; MD: Mediterranean diet; ANT-I: anti-intake; PRO-I: pro-intake; BMI: body mass index; E1–E5: error terms.

Table 3 and Figure 3 present regression weights and standardised regression weights pertaining to the SEM constructed for the overall sample. Outcomes demonstrate that associations exist between ST, SLT, PF, MD, ANT-I, and PRO-I and BMI. Negative relationships were observed between ST and PF (b = −0.137; *p* < 0.005), ST and MD adherence (b = −0.212; *p* < 0.005), ST and SLT (b = −0.221; *p* < 0.005), MD adherence and ANT-I (b = −0.242; *p* < 0.005), ANT-I and PRO-I (b = −0.407; *p* < 0.005), and MD adherence and BMI (b = −0.085; *p* = 0.033). In contrast, positive relationships were found between SLT and MD adherence (b = 0.181; *p* < 0.005), and PRO-I and BMI (b = 0.152; *p* < 0.005).

**Figure 4:**
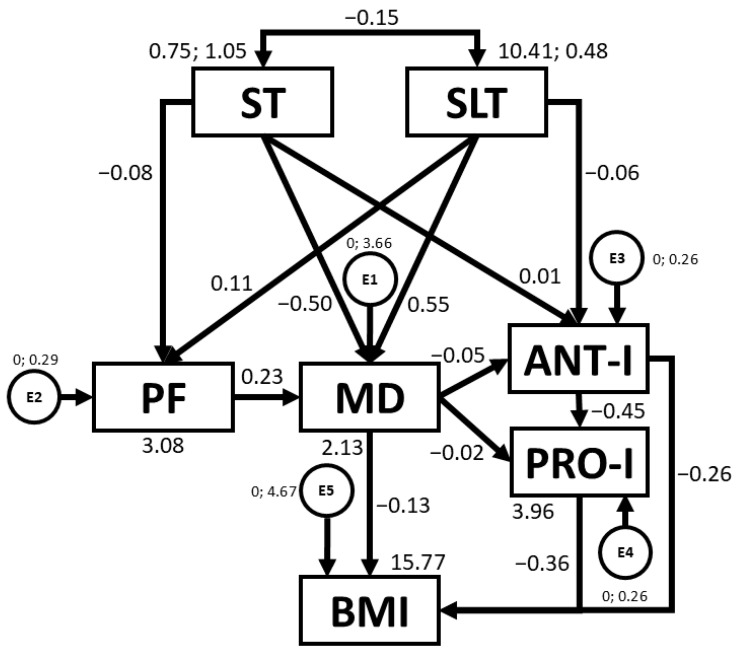
Structural equation model for girls. Note: ST: screen time; SLT: sleep time; PF: physical fitness; MD: Mediterranean diet; ANT-I: anti-intake; PRO-I: pro-intake; BMI: body mass index; E1–E5: error terms.

Table 4 and Figure 4 present regression weights and standardised regression weights pertaining to the SEM developed for girls. The chi-square statistic was non-significant (*χ*^2^ = 3.6; *df* = 7; *p* = 0.821), meaning that, on this occasion, the model could be concluded to exhibit good fit. The model could also be concluded to be homogeneous. Furthermore, NFI, IFI, TLI, and CFI values were excellent, being 0.980, 1.019, 1.063, and 1.000, respectively. At the same time, the RMSEA value was also excellent, being 0.000. This shows that the SEM constructed closely aligns with the empirical data collected, indicating an excellent fit. In this case, negative relationships were observed between ST and PF (b = −0.147; *p* = 0.007), ST and MD adherence (b = −0.259; *p* < 0.005), ST and SLT (b = −0.210; *p* < 0.005), MD adherence and ANT-I (b = −0.175; *p* = 0.002), ANT-I and PRO-I (b = −0.424; *p* < 0.005) ), and MD adherence and BMI (b = −0.114; *p* = 0.036). In contrast, positive relationships were found between SLT and PF (b = 0.137; *p* = 0.012), SLT and MD adherence (b = 0.196; *p* < 0.005), and PRO-I and BMI (b = 0.174; *p* = 0.003).

**Figure 5:**
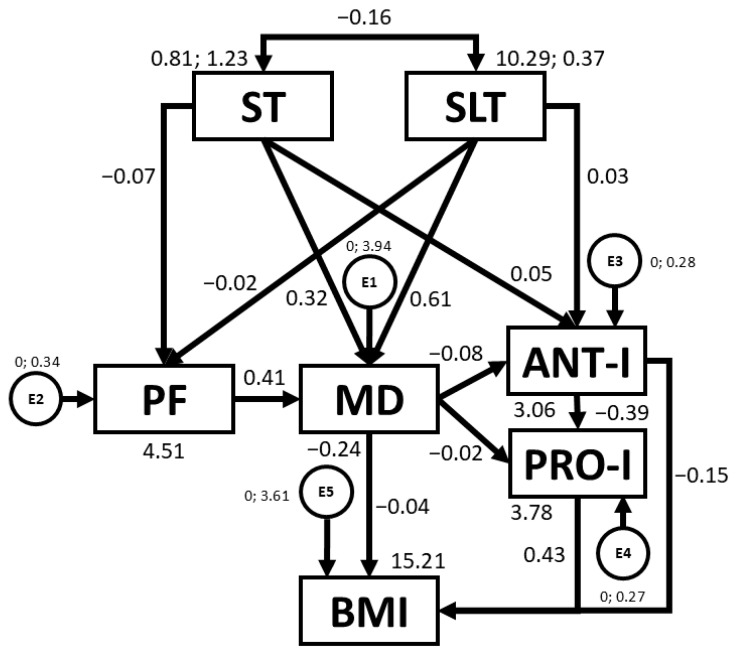
Structural equation model for boys. Note: ST: screen time; SLT: sleep time; PF: physical fitness; MD: Mediterranean diet; ANT-I: anti-intake; PRO-I: pro-intake; BMI: body mass index; E1–E5: error terms.

Table 5 and Figure 5 present the regression weights and standardised regression weights produced by the SEM developed for boys. A statistically significant chi-square outcome was produced (*χ*^2^ = 14.2; *df* = 7; *p* = 0.047). This meant that the null hypothesis was rejected. Thus, other standardised fit indices were used that are less sensitive to sample size. In this sense, NFI and TLI values were acceptable, being 0.912 and 0.906, respectively. Further, IFI and CFI values were excellent, being 0.953 and 0.950, respectively. At the same time, the RMSEA value was acceptable, being 0.057. In this case, negative relationships were observed between ST and PF (b = −0.135; *p* = 0.015), ST and MD adherence (b = −0.170; *p* = 0.002), ST and SLT (b = −0.232; *p* < 0.005), MD adherence and ANT-I (b = −0.302; *p* < 0.005), and ANT-I and PRO-I (b = −0.397; *p* < 0.005). In contrast, positive relationships were found between SLT and MD adherence (b = 0.179; *p* = 0.001), PF and MD adherence (b = 0.114; *p* = 0.035), and PRO-I and BMI (b = 0.124; *p* = 0.039).

**Figure 6:**
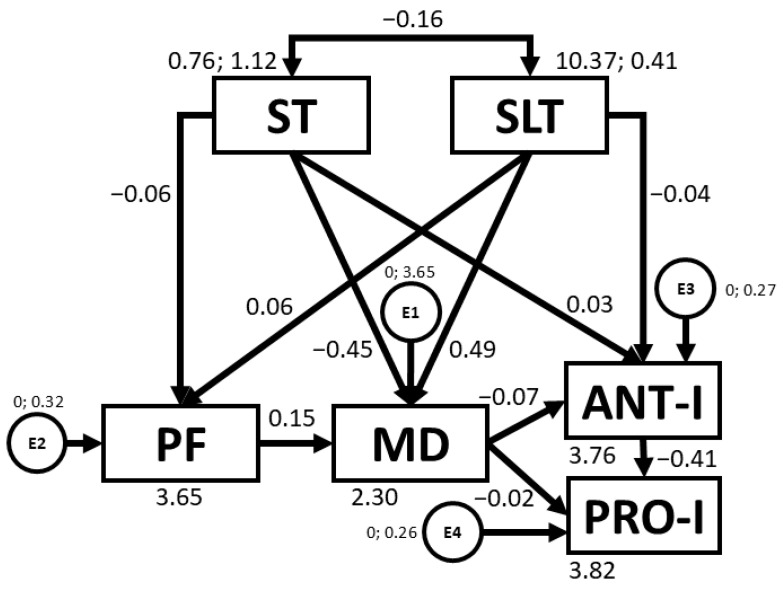
Structural equation model for healthy-weight children. Note: ST: screen time; SLT: sleep time; PF: physical fitness; MD: Mediterranean diet; ANT-I: anti-intake; PRO-I: pro-intake; BMI: body mass index; E1–E5: error terms.

Table 6 and Figure 6 present the regression weights and standardised regression weights produced by the SEM developed for healthy-weight children. The chi-square statistic was statistically significant (*χ*^2^ = 14.8; *df* = 4; *p* = 0.005) meaning that goodness of model fit could not be supported. Thus, other standardised fit indices were used that are less sensitive to sample size. In this sense, NFI, IFI, and CPI values were excellent, being 0.950, 0.961, and 0.959, respectively. TLI and RMSEA values were acceptable, being 0.907 and 0.068, respectively. In this case, negative relationships were observed between ST and PF (b = −0.119; *p* = 0.005), ST and MD adherence (b = −0.234; *p* < 0.005), ST and SLT (b = −0.236; *p* < 0.005), MD adherence and PRO-I (b = −0.078; *p* = 0.049), MD adherence and ANT-I (b = −0.268; *p* < 0.005), and ANT-I and PRO-I (b = −0.417; *p* < 0.005). In contrast, a positive relationship was found between SLT and MD adherence (b = 0.157; *p* < 0.005).

**Figure 7:**
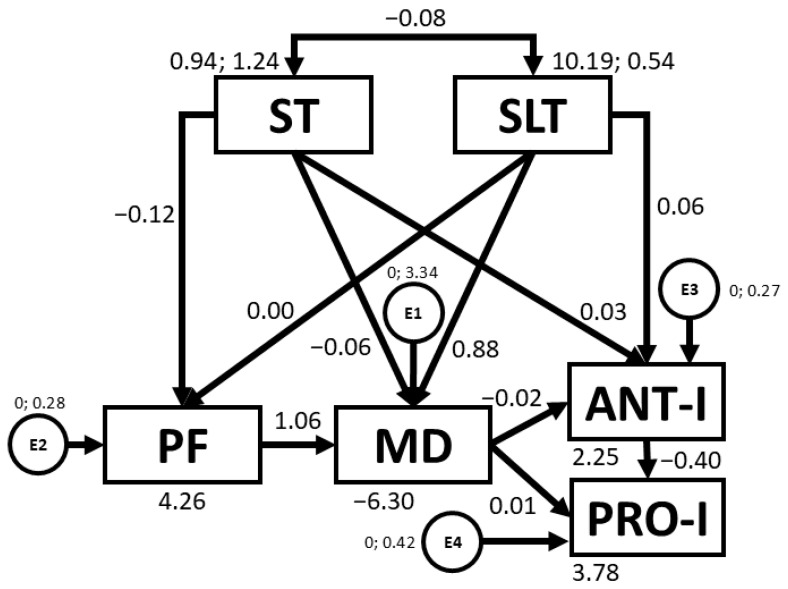
Structural equation model for less healthy-weight children. Note: ST: screen time; SLT: sleep time; PF: physical fitness; MD: Mediterranean diet; ANT-I: anti-intake; PRO-I: pro-intake; BMI: body mass index; E1–E5: error terms.

Table 7 and Figure 7 present the regression weights and standardised regression weights produced by the SEM developed for less healthy-weight children. The chi-square index was non-significant (*χ*^2^ = 0.9; *df* = 4; *p* = 0.930) suggesting that the model was a good fit to the data. The model could also be concluded to be homogeneous. Furthermore, NFI, IFI, TLI, and CFI values were excellent, being 0.971, 1.121, 1.786, and 1.000, respectively. At the same time, the RMSEA value was also excellent, being 0.000. This shows that the SEM created closely aligns with the empirical data collected, indicating an excellent fit. In this case, negative relationships were observed between ST and PF (b = −0.137; *p* = 0.027), and between ANT-I and PRO-I (b = −0.407; *p* = 0.006). In contrast, positive relationships were found between SLT and MD adherence (b = 0.181; *p* = 0.003), and between PF and MD adherence (b = 0.066; *p* = 0.010).

## 4. Discussion

The present research reveals important connections between lifestyle habits and physical and dietary outcomes among pre-schoolers in southern Spain, showing differences influenced by sex and BMI.

Although the topic discussed in the manuscript has been extensively researched, no prior studies have investigated the broader range of potential lifestyle determinants collectively. In this way, the present study accounts for the combined impact of various elements, including ST, SLT, PF, MD adherence, and eating behaviours alongside BMI, allowing for a deeper understanding of interrelationships. The present research takes a novel approach towards examining these variables collectively in pre-school-aged children. Additionally, the study employs measures that have recently been adapted and validated for use with Spanish populations [22].

With regards to present findings, Potter et al. [48] previously reported that PA (r = 0.300; *p* < 0.01) and ST (r = −0.530; *p* < 0.01) might be relevant modifiable correlates of PF in three-year-old pre-schoolers, as revealed by SEM outcomes for the overall sample (b = −0.137, *p* < 0.005) (see Table 3). Targeting both in early childhood interventions is crucial, as reducing ST and increasing PA are effective strategies to improve fitness and health, as shown by associations produced in the present study (*p* < 0.01 for both). Similarly, associations between ST and SLT found by Xu et al. [49] are of a similar magnitude to that found in Table 3. This confirms that children with higher ST exhibit fewer hours of sleep (b = −0.221, *p* < 0.005). In this context, the use of electronic devices before bedtime can disrupt circadian rhythms and increase the risk of sleep disturbances [50]. ST not only affects PF and SLT but also negatively affects MD adherence in the pre-school population, as revealed in Table 3 (b = −0.212, *p* < 0.005). Avery et al. [51] suggest that more ST is often linked to the consumption of high-calorie snacks and unhealthy foods rich in fats and sugars. This combination could directly contribute to an unfavourable increase in BMI and may explain the negative association between MD and BMI reported in Table 3 (b = −0.085, *p* = 0.033). In this sense, lifestyle factors could be the dominant contributors to the early development of obesity [10]. Consequently, pre-school children should be encouraged to move away from watching screens towards engaging in active play, without incurring sleep loss. This is important as sedentary behaviour limits movement and negatively affects PF and MD adherence, aligning with WHO recommendations [13].

In line with MD adherence, Di Nucci et al. [26] observed that PRO-I and ANT- I eating behaviours are incompatible with a healthy diet, as they go hand in hand with failing to consume sufficient amounts of fish (<2–3 servings/week) and typical MD foods such as fruits, vegetables, and legumes and nuts, whilst also consuming high amounts of sweets and fast food. This may explain the negative associations between MD adherence and ANT-I (b = −0.242, *p* < 0.005), and between MD adherence and PRO-I (b = −0.072; *p* = 0.052), detailed in Table 3. At the same time, PRO-I behaviour was positively associated with BMI (b = 0.152, *p* < 0.005). Specifically, the present study found that overweight and obese children exhibited more PRO-I characteristics such as “food responsiveness”, “emotional overeating”, and “enjoyment of food”, alongside fewer ANT-I traits such as reduced “satiety responsiveness” (*p* < 0.05 for all subscales). This is in line with other research which also observed these types of eating behaviours to be higher in children with a less healthy BMI (*p* < 0.001 for all subscales), with no significant outcomes emerging in this group of children with regards to “desire to drink” (*p* = 0.580), as was also revealed in Table 1 (*p* = 0.223) [52]. This suggests that worse behaviours and relationships with healthy food have a negative effect on BMI [22], leading to an increased risk of childhood obesity [53]. Consequently, although most studies on childhood obesity still focus primarily on biological and lifestyle factors, it is also important to consider aspects related to eating behaviour in the development of this disease. Indeed, intervention studies point to the need to pay special attention to parenting behaviour and styles, as well as to the organization of the family eating environment, in order to promote healthy eating behaviour in children and reduce obesity risk [54].

Thus, differential SEM outcomes for healthy weight and overweight/obese children may offer some explanation for the outcomes found regarding BMI (see Table 6 and Table 7).

The aforementioned negative relationship between ST and PF and positive relationships of MD with PF and SLT in relation to the overall sample also emerged in less healthy-weight children (see Table 7). In addition, a positive relationship was also found in this sub-group between PF and MD adherence (b = 0.066, *p* = 0.010). According to Lazarou et al., physical activity mediates the protective effect of MD against childhood obesity, with children with high MD adherence being 83% less likely to be overweight or obese [55]. Thus, interventions targeting less healthy-weight children should promote a more active lifestyle as a means of improving PF [56], whilst, at the same time, reducing exposure to ST. This would likely lead to improved MD adherence and a healthier BMI. Consequently, family involvement and collaboration are key, since parents often introduce their children to screen-based entertainment, even during mealtimes [57]. Furthermore, establishing an early bedtime in combination with lower amounts of ST before bedtime reduces obesity risk and improves diet quality in Spanish children [58].

On the other hand, ST also negatively affects MD adherence and SLT in healthy-weight children (see Table 6). Moreover, it was observed that MD adherence was negatively related to both PRO-I (b = −0.078, *p* = 0.049) and ANT-I (b = −0.268, *p* < 0.005) in these children. Paith et al. [59] previously revealed that fewer PRO-I behaviours, such as those associated with greater satiety response (ANT-I), were also related to less frequent selection of more palatable foods. This helps to control calorie intake and reduce consumption of low-quality processed foods. Such consumption patterns are atypical in terms of the MD and act as precursors to having an overweight or obese weight status. These behaviours are established from the first days of life and can define the way a person eats in the future [54]. Consequently, interventions should be carried out to control eating behaviours in both healthy- and less healthy-weight children, considering biological, environmental and social factors [54].

With regards to sex, SEMs developed for girls and boys revealed a number of similarities and differences with regards to relationships between study variables (see Table 4 and Table 5).

Firstly, given a significant positive relationship between SLT and PF was only observed in girls, an alternative intervention strategy for girls could be to target improved PF. Indeed, according to Latorre-Román et al. [55], girls tend to exhibit lower PF than boys at pre-school age (*p* < 0.05 for all PF test), usually due to the barriers faced by girls to sport participation [60]. Further, the negative relationships between ST and other variables (PF, MD, SLT) were stronger in girls than in boys, particularly in the case of the relationship between ST and MD adherence (b = −0.259, *p* < 0.005 vs. b = −0.212, *p* < 0.005). In this sense, the barriers alluded to earlier may lead girls to adopt more sedentary behaviours during their free time, such as more ST. As previously mentioned, higher ST is often accompanied by the consumption of less healthy foods [17]. Additionally, it is suggested that, particularly in girls, media content often promotes the pursuit of certain beauty standards [61], regardless of whether the diet proposed is healthy or not. Although participants in the present study are young, such messages are typically absorbed from an early age, with girls often imitating behaviours adopted by their mothers [62]. This may also explain why girls in the present study reported greater satiety responsiveness than boys (2.88 ± 0.64 vs. 2.75 ± 0.69, *p* < 0.004) and displayed more ANT-I behaviours (2.94 ± 0.53 vs. 2.86 ± 0.56, *p* < 0.019) (see Table 1). According to Herle [63], eating behaviours are typically learned rather than inherited. Thus, media messages, along with the adoption and imitation of maternal behaviour, which is, in itself, also shaped by subliminal messaging and sociocultural pressures [61], may influence girls from increasingly young ages to conform to beauty standards without considering their health.

Conversely, the fact that PF was associated with MD adherence in boys but not in girls suggests that girls may require additional lifestyle components outside of MD dietary patterns in order to train PF. Boys generally have more opportunities to lead more active lifestyles. In this sense, Stefan et al. [64] reported that more males than females engaged in health-enhancing physical activity (37.5% vs. 18.2%, *p* < 0.001). In order to ensure that preschool girls engage in physical activity at levels comparable to boys, it is vital to create an inclusive environment that counters cultural pressures [60]. This can be achieved by promoting positive role models, emphasising fun over competition, and offering programs tailored to girls’ interests, such as dance or cooperative games [65]. Involving parents can reinforce positive attitudes towards physical activity [66]. Moreover, a promising approach is to promote positive peer relationships and social support from friendship groups in the PA setting [67]. Creating safe and supportive environments for girls to engage in play can enable them to enjoy the health benefits of physical activity in the same way that boys do.

Finally, the use of multi-group analysis based on sex and BMI adds nuance to the findings, whilst the innovative methodology integrates various lifestyle determinants, contributing valuable insights for future research and interventions tailored towards Spanish pre-schoolers. Additionally, the practical implications of present findings are varied and have applications for health, education, and lifestyle interventions. Schools should be the primary venues for such interventions, as they are ideal environments to encourage students and families to foster healthy habits. Indeed, healthy habits learned at school can later be implemented at home and often persist over time [68].

### Limitations, Strengths and Future Perspectives

Present findings should be considered in light of a number of limitations.

One such limitation pertains to the cross-sectional study design employed, which limits the ability make conclusions regarding causal relationships. In future research, longitudinal studies should be considered to analyse the evolution of outcomes.

Moreover, the study’s reliance on self-reported data for various variables increases the risk of measurement error. However, since both the KIDMED for children and the CEBQ have consistently demonstrated strong validity and reliability in similar samples, this issue is unlikely to have substantially influenced findings. Similarly, BMI has known limitations as a stand-alone measure, as it does not account for fat percentage or bone density; however, it remains a practical tool for assessing obesity risk in young children [37]. Specifically, a recent cross-sectional study conducted by Descarpentrie et al. [69] observed that associations between unhealthy lifestyle patterns and BMI may differ depending on the population, with positive relationships only being observed in Spanish and Italian pre-school cohorts. This suggests that BMI may be a good indicator in the context of the present study.

The non-random approach to sample selection should also be considered. Despite this limitation, a large number of participants were recruited from 18 public and private schools in the province of Granada. The large sample size of a difficult to obtain population recruited by the present study provides estimates that better approximate population parameters [70]. In order to boost recruitment, alongside provision of an information letter prior to requesting consent, personal contact details of the principal investigator were provided to participants. This enabled parents to request additional information prior to study start, helped to foster trust in researchers, and promoted parental motivation to participate. In addition, participants were informed throughout the study process that they could withdraw from the study at any time. These measures were established following suggestions made by Sammons et al. [71] for motivating parents to consent to participation.

Another limitation is that the parents of participating pre-schoolers withheld their consent for socioeconomic status data to be accessed. Future research can/should investigate the influence of parents’ healthy habits on their children, whilst considering other sociocultural factors such as the family’s education or socioeconomic status, as these appear to be key factors when it comes to promoting healthy habits in pre-school-aged children. Although many potential cofounders were controlled for, some physiological and genetic factors may have still confounded estimations. In addition, it would be interesting for future research to evaluate other variables that may be related to healthy lifestyles such as cognitive performance, mental health, and general well-being.

Furthermore, it must be acknowledged that, to the best of the authors’ knowledge, the present study represents one of very few studies available that conduct integrative analysis of various healthy lifestyle determinants in children under six years of age. Thus, further scientific research is required at this stage.

## 5. Conclusions

Pre-schoolers from southern Spain with lower ST tend to have higher PF, MD adherence, and SLT. Likewise, pre-schoolers who report adequate MD adherence exhibit more SLT, fewer PRO-I and ANT-I behaviours, and a lower BMI. With regards to the latter, children with a higher BMI exhibit more PRO-I behaviours. Relationships between these factors are clearer in girls and pre-schoolers who are overweight or obese, indicating that both sex and weight status play a role in these interactions.

In order to improve PF in overweight or obese children, interventions should focus on reducing ST. As indicated by the correlations found in the present study, such interventions are crucial given that good PF is likely to lead to better MD adherence in pre-schoolers. This, in turn, will bring about reductions in BMI and reduce the risk of being overweight or obese. Specifically in the case of girls, it is suggested that interventions focus on reducing ST and increasing SLT as a means of improving both PF and MD adherence. Whilst this strategy is also likely to be beneficial for boys, the promotion of MD adherence by targeting PF will likely produce better outcomes.

## Figures and Tables

**Table 1:** Descriptive characteristics of the study sample according to sex and BMI.

		Sex			BMI		
	Overall (*n* = 653)	Male (*n* = 317)	Female (*n* = 336)	*p*	Healthy Weight (*n* = 580)	Overweight/ Obese (*n* = 73)	*p*
Age (years)	4.78 ± 0.93	4.78 ± 0.97	4.78 ± 0.90	0.892	4.76 ± 0.93	4.92 ± 0.92	0.210
BMI (kg/m^2^)	15.53 ± 2.09	15.52 ± 1.92	15.53 ± 2.23	0.691			
ST (hours)	1.78 ± 1.07	1.81 ± 1.11	1.75 ± 1.02	0.604	1.76 ± 1.06	1.94 ± 1.12	0.271
SLT (hours)	10.35 ± 0.66	10.29 ± 0.61	10.41 ± 0.70	0.011	10.37 ± 0.64	10.19 ± 0.74	0.147
MD adherence	7.17 ± 2.03	7.18 ± 2.09	7.16 ± 1.97	0.999	7.21 ± 2.02	6.79 ± 2.05	0.115
Overall PF	4.11 ± 0.57	4.14 ± 0.59	4.08 ± 0.55	0.104	4.12 ± 0.57	4.01 ± 0.55	0.069
GPF	4.28 ± 0.65	4.30 ± 0.067	4.26 ± 0.62	0.267	4.30 ± 0.64	4.14 ± 0.65	0.045
CF	4.05 ± 0.75	4.09 ± 0.76	4.02 ± 0.74	0.203	4.08 ± 0.75	3.82 ± 0.71	0.003
MS	4.02 ± 0.70	4.06 ± 0.71	3.99 ± 0.69	0.223	4.03 ± 0.69	4.01 ± 0.75	0.902
SA	4.12 ± 0.72	4.17 ± 0.72	4.07 ± 0.73	0.074	4.13 ± 0.74	4.05 ± 0.62	0.237
B	4.07 ± 0.71	4.08 ± 0.74	4.07 ± 0.69	0.765	4.08 ± 0.72	4.05 ± 0.62	0.495
PRO-I	2.51 ± 0.56	2.50 ± 0.56	2.51 ± 0.56	0.998	2.48 ± 0.53	2.74 ± 0.68	0.003
FR	2.22 ± 0.84	2.17 ± 0.80	2.27 ± 0.87	0.258	2.18 ± 0.80	2.54 ± 1.06	0.008
EO	1.76 ± 0.64	1.74 ± 0.65	1.78 ± 0.64	0.442	1.73 ± 0.62	1.97 ± 0.77	0.015
EF	3.59 ± 0.80	3.60 ± 0.82	3.58 ± 0.78	0.407	3.56 ± 0.80	3.83 ± 0.74	0.006
DD	2.46 ± 0.87	2.48 ± 0.91	2.43 ± 0.84	0.560	2.44 ± 0.86	2.61 ± 0.96	0.223
ANT-I	2.90 ± 0.55	2.86 ± 0.56	2.94 ± 0.53	0.019	2.92 ± 0.56	2.77 ± 0.53	0.023
SR	2.82 ± 0.67	2.75 ± 0.69	2.88 ± 0.64	0.004	2.84 ± 0.67	2.66 ± 0.67	0.037
SE	3.08 ± 0.79	3.02 ± 0.81	3.13 ± 0.77	0.050	3.10 ± 0.79	2.92 ± 0.71	0.093
EU	2.96 ± 0.60	2.94 ± 0.60	2.98 ± 0.60	0.429	2.98 ± 0.59	2.86 ± 0.61	0.129
FF	2.76 ± 0.84	2.73 ± 0.86	2.80 ± 0.81	0.193	2.78 ± 0.84	2.65 ± 0.81	0.211

Note: BMI: body mass index; ST: screen time; SLT: sleep time; MD: Mediterranean diet; PF: physical fitness; GPF: general physical fitness; CF: cardiorespiratory fitness; MS: muscular strength; SA: speed/agility; B: balance; PRO-I: pro-intake; FR: food responsiveness; EO: emotional overeating; EF: enjoyment of food; DD: desire to drink; ANT-I: anti-intake; SR: satiety responsiveness; SE: slowness in eating; EU: emotional undereating; FF: food fussiness.

**Table 2:** Correlation coefficients produced between study variables adjusted for sex.

	BMI	ST	SLT	Overall PF	MD	ANT-I
ST	0.074					
SLT	−0.053	−0.219 **				
Overall PF	−0.025	−0.152 **	0.093 *			
MD	−0.062	−0.262 **	0.235 **	0.113 *		
ANT-I	−0.105 **	0.144 **	−0.106 **	−0.160 **	−0.287 **	
PRO-I	0.178 **	0.002	0.015	0.067	0.039	−0.403 **

Note: * p < 0.05; ** *p* < 0.01. BMI: body mass index; ST: screen time; SLT: sleep time duration; PF: physical fitness; MD: Mediterranean diet; ANT-I: anti-intake; PRO-I: pro-intake.

**Table 3:** Regression weights for the overall sample.

Association Between Variables	RW				SRW
	Estimation	SE	CR	*p*	Estimation
PF ← ST	−0.073	0.021	−3.466	***	−0.137
PF ← SLT	0.050	0.034	1.451	0.147	0.058
MD ← SLT	0.559	0.117	4.764	***	0.181
MD ← PF	0.233	0.133	1.750	0.080	0.066
MD ← ST	−0.403	0.073	−5.537	***	−0.212
ANT-I ← ST	0.032	0.020	1.554	0.120	0.062
ANT-I ← SLT	−0.012	0.033	−0.379	0.705	−0.015
ANT-I ← MD	−0.066	0.011	−6.088	***	−0.242
PRO-I ← ANT-I	−0.415	0.038	−10.924	***	−0.407
PRO-I ← MD	−0.020	0.010	−1.941	0.052	−0.072
BMI ← ANT-I	−0.264	0.165	−1.606	0.108	−0.069
BMI ← PRO-I	0.566	0.156	3.632	***	0.152
BMI ← MD	−0.088	0.041	−2.130	0.033	−0.085
SLT ↔ ST	−0.155	0.028	−5.508	***	−0.221

Note: PF: physical fitness; ST: screen time; SLT: sleep time; MD: Mediterranean diet; ANT-I: anti-intake; PRO-I: pro-intake; BMI: body mass index; RW: regression weight; SRW: standardised regression weight; ←: directional relationships between variables; ↔: correlation between two variables. *** p < 0.005.

**Table 4:** Regression weights produced by the SEM developed for girls.

Association Between Variables	RW				SRW
	Estimation	SE	CR	*p*	Estimation
PF ← ST	−0.079	0.029	−2.693	0.007	−0.147
PF ← SLT	0.109	0.043	2.523	0.012	0.137
MD ← SLT	0.553	0.149	3.716	***	0.196
MD ← PF	0.034	0.186	0.185	0.853	0.010
MD ← ST	−0.497	0.101	−4.908	***	−0.259
ANT-I ← ST	0.011	0.029	0.395	0.693	0.022
ANT-I ← SLT	−0.056	0.042	−0.1324	0.185	−0.074
ANT-I ← MD	−0.047	0.015	−3.067	0.002	−0.175
PRO-I ← ANT-I	−0.448	0.054	−8.351	***	−0.424
PRO-I ← MD	−0.018	0.014	−1.256	0.209	−0.064
BMI ← ANT-I	−0.361	0.251	−1.436	0.151	−0.085
BMI ← PRO-I	−696	0.233	2.988	0.003	0.174
BMI ← MD	−0.129	0.061	−2.094	0.036	−0.114
SLT ↔ ST	−0.149	0.040	−3.764	***	−0.210

Note: PF: physical fitness; ST: screen time; SLT: sleep time; MD: Mediterranean diet; ANT-I: anti-intake; PRO-I: pro-intake; BMI: body mass index; RW: regression weight; SRW: standardised regression weight; ←: directional relationships between variables; ↔: correlation between two variables. *** p < 0.005.

**Table 5:** Regression weights produced by the SEM developed for boys.

Association Between Variables	RW				SRW
	Estimation	SE	CR	*p*	Estimation
PF ← ST	−0.071	0.030	−2.361	0.018	−0.135
PF ← SLT	−0.024	0.055	−0.426	0.570	−0.024
MD ← SLT	0.614	0.189	3.241	0.001	0.179
MD ← PF	0.406	0.193	2.108	0.035	0.114
MD ← ST	−0.319	0.104	−3.058	0.002	−0.170
ANT-I ← ST	0.054	0.028	1.925	0.054	0.107
ANT-I ← SLT	0.028	0.052	−544	0.587	0.030
ANT-I ← MD	−0.082	0.015	−5.469	***	−0.302
PRO-I ← ANT-I	−0.393	0.054	−7.216	***	−0.397
PRO-I ← MD	−0.021	0.015	−1.446	0.148	−0.079
BMI ← ANT-I	−0.154	0.215	−0.715	0.474	−0.045
BMI ← PRO-I	0.427	0.206	2.069	0.039	0.124
BMI ← MD	−0.043	0.054	−0.790	0.430	−0.047
SLT ↔ ST	−0.156	0.039	−4.021	***	−0.232

Note: PF: physical fitness; ST: screen time; SLT: sleep time; MD: Mediterranean diet; ANT-I: anti-intake; PRO-I: pro-intake; BMI: body mass index; RW: regression weight; SRW: standardised regression weight; ←: directional relationships between variables; ↔: correlation between two variables. *** p < 0.005.

**Table 6:** Regression weights produced by the SEM developed for healthy-weight children.

Association Between Variables	RW				SRW
	Estimation	SE	CR	*p*	Estimation
PF ← ST	−0.064	0.023	−2.819	0.005	−0.119
PF ← SLT	0.056	0.038	1.496	0.135	0.063
MD ← SLT	0.491	0.127	3.855	***	0.157
MD ← PF	0.147	0.141	1.043	0.297	0.042
MD ← ST	−0.446	0.078	−5.736	***	−0.234
ANT-I ← ST	0.030	0.022	1.396	0.163	0.059
ANT-I ← SLT	−0.036	0.035	−1.010	0.313	−0.042
ANT-I ← MD	−0.073	0.011	−6.408	***	−0.268
PRO-I ← ANT-I	−0.406	0.039	−10.485	***	−0.417
PRO-I ← MD	−0.021	0.011	−1.966	0.049	−0.078
SLT ↔ ST	−0.161	0.029	−5.529	***	−0.236

Note: PF: physical fitness; ST: screen time; SLT: sleep time; MD: Mediterranean diet; ANT-I: anti-intake; PRO-I: pro-intake; RW: regression weight; SRW: standardised regression weight; ←: directional relationships between variables; ↔: correlation between two variables. *** p < 0.005.

**Table 7:** Regression weights produced by the SEM developed for less healthy-weight children.

Association Between Variables	RW				SRW
	Estimation	SE	CR	*p*	Estimation
PF ← ST	−0.123	0.056	−2.206	0.027	−0.137
PF ← SLT	−0.001	0.085	−0.014	0.989	0.058
MD ← SLT	0.880	0.294	2.991	0.003	0.181
MD ← PF	1.058	0.410	2.579	0.010	0.066
MD ← ST	−0.057	0.201	−0.283	0.777	−0.212
ANT-I ← ST	0.028	0.055	0.506	0.613	0.062
ANT-I ← SLT	0.062	0.088	0.699	0.485	−0.015
ANT-I ← MD	0.024	0.032	−0.749	0.454	−0.242
PRO-I ← ANT-I	−0.398	0.146	−2.725	0.006	−0.407
PRO-I ← MD	0.009	0.068	−0.233	0.815	−0.072
SLT ↔ ST	−0.078	0.097	−0.809	0.419	−0.221

Note: PF: physical fitness; ST: screen time; SLT: sleep time; MD: Mediterranean diet; ANT-I: anti-intake; PRO-I: pro-intake; RW: regression weight; SRW: standardised regression weight; ←: directional relationships between variables; ↔: correlation between two variables.

## Data Availability

Data are available from the authors on request. Data are not publicly available due to privacy reasons.
